# Assessment of Biofilm Formation by *Candida albicans* Strains Isolated from Hemocultures and Their Role in Pathogenesis in the Zebrafish Model

**DOI:** 10.3390/jof8101014

**Published:** 2022-09-27

**Authors:** Sabi Pokhrel, Nawarat Boonmee, Orawan Tulyaprawat, Sujiraphong Pharkjaksu, Iyarit Thaipisutikul, Phoom Chairatana, Popchai Ngamskulrungroj, Chalermchai Mitrpant

**Affiliations:** 1 Department of Microbiology, Faculty of Medicine Siriraj Hospital, Mahidol University, Bangkok 10700, Thailand; 2 Department of Biochemistry, Faculty of Medicine Siriraj Hospital, Mahidol University, Bangkok 10700, Thailand

**Keywords:** biofilm, *Candida albicans*, gene, virulence, zebrafish

## Abstract

*Candida albicans*, an opportunistic pathogen, has the ability to form biofilms in the host or within medical devices in the body. Biofilms have been associated with disseminated/invasive disease with increased severity of infection by disrupting the host immune response and prolonging antifungal treatment. In this study, the *in vivo* virulence of three strains with different biofilm formation strengths, that is, non-, weak-, and strong biofilm formers, was evaluated using the zebrafish model. The survival assay and fungal tissue burden were measured. Biofilm-related gene expressions were also investigated. The survival of zebrafish, inoculated with strong biofilms forming *C. albicans,*, was significantly shorter than strains without biofilms forming *C. albicans.* However, there were no statistical differences in the burden of viable colonogenic cell number between the groups of the three strains tested. We observed that the stronger the biofilm formation, the higher up-regulation of biofilm-associated genes. The biofilm-forming strain (140 and 57), injected into zebrafish larvae, possessed a higher level of expression of genes associated with adhesion, attachment, filamentation, and cell proliferation, including *eap1*, *als3*, *hwp1*, *bcr1*, and *mkc1* at 8 h. The results suggested that, despite the difference in genetic background, biofilm formation is an important virulence factor for the pathogenesis of *C. albicans*. However, the association between biofilm formation strength and *in vivo* virulence is controversial and needs to be further studied.

## 1. Introduction

The yeast *Candida albicans* is part of the microbiota of healthy individual’s oral, genital, and gastrointestinal tracts. The rate of *Candida* carriage on the mucosal surface in healthy individual ranges from 30% to 70% [1]. However, several factors, such as extreme age, pregnancy, chemotherapy, and cancer or prolonged use of broad-spectrum antibiotics and antifungal agents, which cause immune suppression, enable *Candida* to invade host tissue and cause mild superficial to severe systemic infection [2,3,4]. During this process, virulence characteristics, namely, the transition between yeast and hyphal forms; biofilm formation; the secretion of enzymes such as phospholipases and proteinases; the expression of adhesins and invasins on the cell surface; and phenotypic switching, are crucial to establishing infection [5].

The biofilm formed by *Candida* spp. is a complex community of yeast cells, hyphal forms, and pseudohyphal forms surrounded by an extracellular matrix of polysaccharides, proteins, lipids, and DNA [6]. Previous studies reported that approximately 80% of *C. albicans* infections in humans are directly or indirectly involved with biofilm formation on the host body or abiotic surfaces, including indwelling medical devices, such as vascular catheters, prosthetic heart valves, joint replacements, or dental implants. This biofilm mostly results in high morbidity and mortality [7,8].

Biofilm formation involves a series of events that begin with attachment/adhesion, the formation of basal microcolonies, and hyphal filamentation to release cells from the complex [9]. Host immune cells respond differently to *Candida* spp. when under planktonic conditions [10]. The biofilm matrix is partially protected from neutrophil activities, including phagocytosis, degranulation, and the formation of neutrophil extracellular traps (NET) [11]. Furthermore, cells that detach from such biofilms are more cytotoxic and increase host mortality, as seen in the murine model study [12].

In addition, biofilm formation is one of the main factors mitigating the effectiveness of antifungal treatments. As biofilm is typically a group of densely populated yeast cells surrounded by an extracellular matrix, this structure leads to a reduction in the effective drug concentration for the cells within the biofilm. A previous study found that the sessile cells within biofilm structure is more resistant to antifungal drugs than their planktonic cells [13].

Previous studies reported that among the different clinical isolates of *Candida*, biofilm formation can vary [14,15]. However, the effect on virulence of the ability to form biofilms between different strains of *C. albicans* using the zebrafish infection model is not known. Therefore, this study aims to compare virulence between biofilm and non-biofilm forming clinical *C. albicans* isolates using the zebrafish model of infection with associated gene expression for biofilm production.

## 2. Materials and Methods

### 2.1. Fungal Strains and Their In Vitro Virulence Study

The commercially available laboratory strain of *C. albicans* (ATCC 90028) was used as a positive control for biofilm formation. Other strains of *C. albicans* 104, 57, and 140, used in this study, were isolated from hemoculture in the Department of Microbiology, Faculty of Medicine Siriraj Hospital, Mahidol University, Thailand. Species identification was confirmed by both phenotypic and genotypic methods.

At the beginning, all *C. albicans* strains collected from positive hemocultures during April 2016 and November 2017 were evaluated for their four *in vitro* virulence activities, comprising phospholipase activity, proteinase activity, hemolytic activity, and biofilm formation, as previously described [16]. Of 46 *C. albicans* blood strains, the three isolates, *C. albicans* 104, 57, and 140, producing different biofilm levels but the same pattern of the other three virulence results, were divided into four *in vitro* virulence factors.

To confirm the four *in vitro* virulence factors, i.e., comprising phospholipase activity, proteinase activity, hemolytic activity, and biofilm formation. The isolates were pre-grown on Sabouraud Dextrose Agar (SDA) at 37 °C for 48 h before being suspended in a phosphate buffer saline (PBS) pH 7.4 to produce a 0.5 McFarland standard (1–5 × 10^6^ cells/mL of yeast suspension). The 5 µL suspension was inoculated onto egg yolk medium, bovine serum albumin, and blood agar to determine phospholipase, proteinase, and hemolytic activity, respectively.

The phospholipase activity was performed by measuring the size of the precipitation zone after growth on egg yolk agar at 37 °C for 48 h [17]. The value of phospholipase activity (Pz) was determined by the ratio of the diameter of the colony to the total diameter of the colony plus the visible precipitation zone. A Pz value of 1.00 indicates no phospholipase activity, and less than 1.00 shows phospholipase activity. The lower the Pz value, the higher the enzymatic activity. Reference strains of *C. albicans* (ATCC 24433) served as a positive control.

Proteinase activity was analyzed using the presence of a clear halo zone around a five-days colony grown at 37 °C on a bovine serum albumin medium. The clear halo zone around the colony was recorded as evidence of proteinase activity, and the level of activity was determined by the ratio of the colony diameter to the total diameter of the colony plus the halo zone, Pr value. A Pr value of 1.00 indicates no proteinase activity, and less than 1.00 shows proteinase activity [18]. Reference strains of *C. albicans* (ATCC 10231) served as a positive control.

For determination of hemolytic activity, the tested and control strains on SDA supplemented with 3% enriched glucose and 6.5% fresh sheep blood were examined after incubation at 37 °C for 48 h. The presence of a distinct translucent halo around the yeast colony indicated positive hemolytic activity. The ratios of the diameter of the colony to the total diameter of the colony plus the translucent halo zone (Hz value) were used to represent the level of hemolytic activity as follows: Hz < 0.64, strongly positive; 0.64 ≤ Hz < 1.00, positive; and Hz = 1. Reference strains of *C. albicans* (ATCC 90028) served as a positive control [17,18].

For the assessment of biofilm formation, 2,3-Bis-(2-Methoxy-4-Nitro-5-Sulfophenyl)-2H-Tetrazoilum-5-Carboxanilide (XTT) reduction assay (Thermo Scientific, Waltham, MA, USA) using 96-well microplates was used. Briefly, an 18 h culture of *C. albicans* strains was adjusted to a 0.5 McFarland standard with yeast extract peptone dextrose (YPD) medium. The yeast suspensions (200 µL) were seeded in flat bottom 96-well plates [19]. After sealing, the plates were incubated at 37 °C for 48 h. The wells were washed with 200 μL PBS three times followed by the addition of a 100 µL mixture of 1 mg/mL of XTT and 10 µL of activation reagent, phenazine methosulfate, to the wells. The plates were further incubated in the dark for 3 h, and the biofilms were quantified at A450 and A630 using a plate reader. Microtitre wells containing only YPD medium that were not inoculated were used as negative controls. The cut-off optical density (ODc) was defined as three standard deviations above the mean OD of the negative control, and the strains were classified as follows: OD ≤ ODc = no biofilm producer; ODc < OD ≤ 2 × ODc = weak biofilm producer; 2 × ODc < OD ≤ 4 × ODc = moderate biofilm producer; and 4 × ODc < OD = strong biofilm producer [20,21,22,23]. Reference strains of *C. albicans* (ATCC 90028) served as a positive control strain, while *C. glabrata* (ATCC 90030) was used as a negative control strain.

### 2.2. Microinjection in Zebrafish Larvae

*C. albicans* strains were grown on YPD agar at 30 °C. The liquid cultures were grown for 18 h in 5 mL of YPD broth in a shaking incubator at 250 rpm at 30 °C for injections. Yeast cells were centrifuged at 3000× *g*, washed twice with PBS, and resuspended in 2 mL of PBS. The concentration of the yeast suspension was diluted to provide an OD 600 nm of 0.7 (~10^7^ cells/mL). This diluted suspension was resuspended in autoclaved 5% polyvinylpyrrolidone 40 (PVP40) with 0.5% phenol red in PBS [24]. The PBS containing 5% PVP40 and 0.5% phenol red was used as a control.

Five days after fertilization, larvae of the wild-type zebrafish strain (AB strain) were used for *Candida* infection experiments. The larvae were anesthetized in E3-MB, containing 4 mg/mL of tricaine (ethyl-1,3-aminobenzoate; Sigma-Aldrich, St. Louis, MO, USA). The yeast cells were inoculated into zebrafish larvae using a microinjector. Two to three nanoliters of the yeast suspension was injected into the swim bladder of the larvae. The control and yeast-infected larvae were kept in an E3 buffer at 30 °C. This study was approved by the Siriraj Animal Care and Use Committee (SiACUC) (020/2562; 1 March 2022).

### 2.3. Survival Study

The survival study of zebrafish larvae was carried out in the control group and the yeast-infected group using 25 larvae per group. The larvae from each group were daily checked for their heartbeat under the light stereomicroscope. No heartbeat of zebrafish larvae observed was considered as lethal endpoint. Survival was recorded daily for up to nine days. The data were subsequently evaluated with the Mantel–Cox test using GraphPad Prism 8.

### 2.4. Fungal Tissue Burden/CFU Assay

The infectivity in the larvae was assessed by quantification of the colony forming unit (CFU) using the spread plate technique in biological triplications. Five representative larvae were pooled and homogenized at 8 hpi (hours post infection), 24 hpi, and 96 hpi in 100 μL of 0.85% normal saline. For plating, 100 μL of homogenate from each group was plated on SDA agar supplemented with 50 mg/L chloramphenicol. Undiluted homogenate, 1:10, 1:100, and 1:1000 dilutions were plated for the sample in duplicate plates at each time point. The plates were incubated at 30 °C for 48 h for the counting of CFU [24,25].

### 2.5. RNA Extraction and Gene Expression Analysis

RNA was isolated from larvae 4, 8, 12, 24, and 96 h post infection (hpi) using TRIzol reagent. To synthesize cDNA, a total of 2 µg of RNA was used in a reverse transcription reaction using Super Script III RT (Life Technologies, Carlsbad, CA, USA) and a random decamer according to the manufacturer’s instructions or iScript Transcriptase (Bio-Rad, Hercules, CA, USA). The genes of interest and the primer pairs used are provided in the table below (Table 1). Real-time PCR was performed on a Roche Light Cycler-480. cDNA amplification was performed with an initial denaturation at 95 °C for 10 min followed by 40 cycles of denaturation (95 °C for 10 s), annealing (50–54 °C for 30 s), and extension (72 °C for 20 s). This was followed by a melting curve generation and cooling time. The determination of gene expression was made by calculating the normalized expression ratio (2^−ΔΔCt^). In brief, data from the comparative expression approach were produced as fold changes in gene expression relative to a control or baseline condition, normalized to the constitutive reference gene *act1*. In this case, the baseline condition was mRNA isolated from planktonically grown *C. albicans* of the corresponding strains [24,26].

### 2.6. Statistical Analysis

The statistical analyses were performed using GraphPad Prism 8. CFU quantification and normalized gene expressions at different time points and were compared by ordinary one-way or two-way ANOVA with Tukey’s multiple comparison test according to the requirements. In all cases, a *p*-value less than 0.05 was considered significant.

## 3. Results

### 3.1. In Vitro Virulence Assessment

Among the isolates of *C. albicans* strains obtained from hemoculture in the hospital laboratory, the three isolates with mainly differences in biofilm activity were selected for the study of biofilm formation *in vitro* and *in vivo* animal models. *C. albicans* strains 104, 57, and 140 with similar phospholipase activity, proteinase activity, and hemolytic activity (Table 2) were selected.

### 3.2. Survival Assay

**Figure 1:**
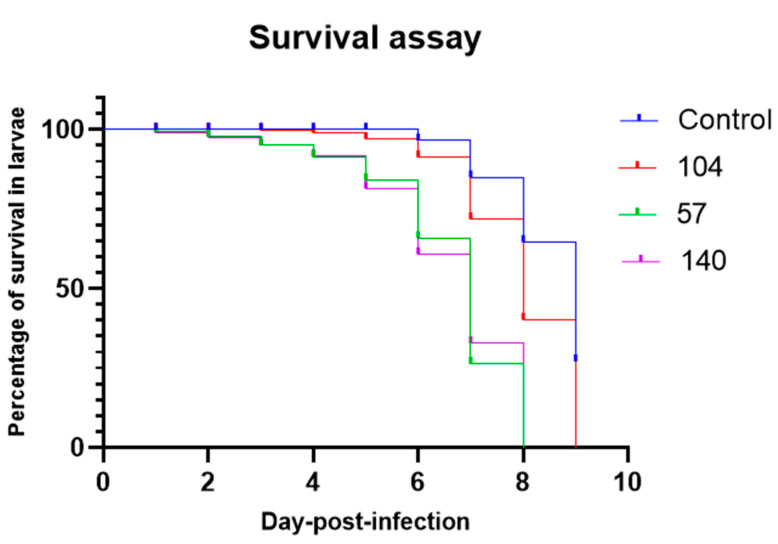
Survival of zebrafish larvae injected with either *C. albicans* strains 104 (non-biofilm former), 57 (weak biofilm former), or 140 (strong biofilm former). The control group was injected with PBS without an organism. Each experiment was performed in duplicate.

The survival of zebrafish larvae infected with different strains of *C. albicans* was monitored for nine days post infection (Figure 1). Deaths in the group of zebrafish larvae infected with all three strains of *C. albicans* were statistically significantly different (*p* < 0.0001) when compared to the control group. Similarly, there was statistically significant (*p* < 0.0001) more death in zebrafish infected with biofilm-forming strain (140 and 57) compared to non-biofilm strain (104). However, there were no significant differences (*p* > 0.05) between larvae infected with weak biofilm strains (57) and strong biofilm strains (140).

### 3.3. Tissue Burden Assessment

**Figure 2:**
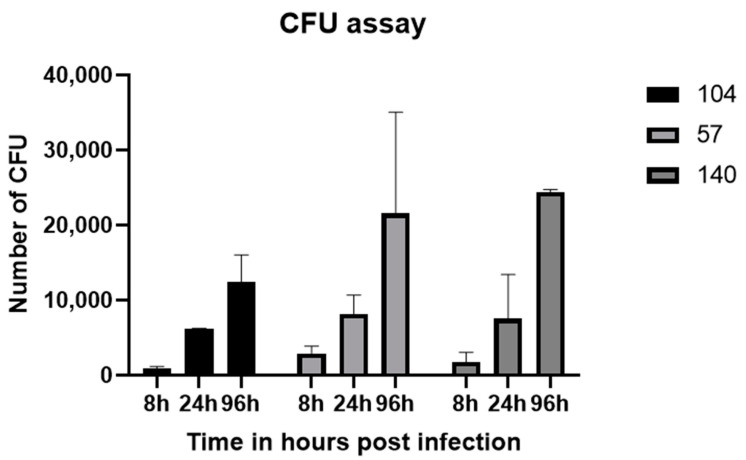
Fungal tissue burden in zebrafish larvae that were infected with yeast cells of *C. albicans* strains 104, 57, or 140. Each experiment was performed in triplicate. CFU, colony forming unit.

As demonstrated in Figure 2, the number of *C. albicans* cells showed an increasing pattern from 8 to 96 hpi, which indicated cell proliferation. However, no significant differences were observed between strains at any time point.

### 3.4. Gene Expression Related to Biofilm Genes

**Figure 3:**
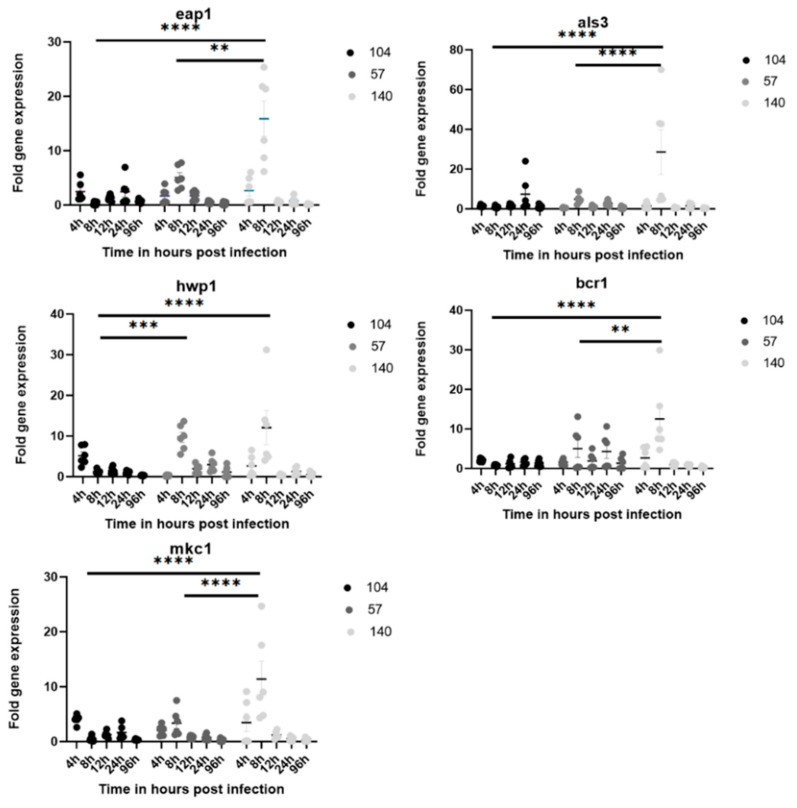
The expression levels of *eap1*, *als3*, *hwp1*, *bcr1*, and *mkc1* in *C. albicans strains* 104, 57, and 140 infected zebrafish larvae, by time point. Each experimental group of 30 zebrafish was injected with 0.7 OD yeast cells of one of the *C. albicans* strains. The normalized ratios of expression were calculated by comparison with the level of expression of the *act-1* gene in each group of planktonic cells. Each experiment was performed in duplicate. h, hour; **, *p* < 0.01; ***, *p* < 0.001; and ****, *p* < 0.0001.

For the determination of biofilm activity within host cells, the expression profiles of the genes that played an important role in biofilm formation during *C. albicans* infection were monitored by real-time quantitative PCR (qPCR) 4 hpi (very early), 8 hpi (early), 12 hpi (intermediate), 24 hpi (late), and 96 hpi (very late). Generally, gene up-regulations were mostly seen at 8 hpi (Figure 3). It was observed that all genes (*eap1*, *als3*, *hwp1*, *bcr1*, and *mkc1*) in this study were significantly up-regulated in the strong biofilm-forming strain (140) compared to the non-biofilm strain (104) at 8 hpi. Similarly, there was a significant difference in gene expression between the weak biofilm strain (57) and the strong biofilm strain (140) for four of the above genes, except *hwp1*. No significant differences were observed in gene expression between different strains at the other corresponding time points (4, 12, 24, and 96 hpi).

## 4. Discussion

*C. albicans* is considered to be the strongest biofilm producer among *Candida* species [30,31]. The biofilm provides the fungus with greater adaptation in the host cell with protection against immune defenses and antifungal agents and is a reservoir for persistent infections [32,33]. In this study, more deaths in zebrafish infected with the strong biofilm former occurred as soon as 24 hpi, compared to late deaths in the strain without biofilm formation. However, zebrafish infected with biofilm-forming strains did not show significantly higher fungal burdens in tissue compared to non-biofilm-forming strains. Strong biofilm-forming strains showed more up-regulation of biofilm-related genes such as *eap1*, *als3*, *hwp1*, *bcr1*, and *mkc1* eight hours after infection in the zebrafish model.

As expected, the strong biofilm former was more virulent in the zebrafish model of infection despite the difference in genetic background. A previous study by Pham LTT et al. showed different clinical outcomes (death or recovery) in the human patient infected with different strains of *C. albicans* with higher mortality for those infected with high biofilm-forming strains [16]. In another study with the *Galleria mellonella* model of infection, high biofilm formation mortality was significantly increased in comparison to low biofilm mortality (*p* = 0.0005) [14]. Similar studies in the past identified that the classification of clinical isolates as high or low biofilm formation abilities is a good predictor of clinical outcomes as high biofilm formers have been shown to correlate significantly with mortality [14,34].

Interestingly, despite the more *in vivo* virulence of the strong biofilm former, no statistically significant differences in fungal tissue burden were observed. This was similar to the CFU quantification assay we performed by scrapping the biofilm wells after washing at 48 h, where there were also no significant differences between CFU between biofilm and non-biofilm strains. This may be possible due to the increased proliferation of non-biofilm strains (lower doubling time) even when adhered to a low number compared to the biofilm, which is reciprocal to a study in which slow grower *C. albicans* exhibited higher biofilm formation [31].

During the steps of biofilm formation, several genes have been associated with each step that helps the fungus establish a well-organized biofilm system. Genes such as *eap1* and *als3* associated with the initial attachment of yeast cells to the surface were found to be significantly up-regulated in strains forming high biofilms during infection in zebrafish at the initial time point of 8 hpi. *Hwp1*, which encodes the cell wall mannoprotein of the hyphal forms and the germ tube and is found to be strongly induced during hyphal growth, was up-regulated at 8 hpi in our study.

This suggests that this time point is suitable for filamentation and increasing fungal biomass in the biofilm as per our study. In congruence with our study, *als3* and *hwp1* were found to be highly expressed in vaginal isolate biofilm strains in a previous study [35]. Similarly, a previous study by Nailis et al. reported that *hwp1* and *als3* are constitutively expressed in *C. albicans* biofilms in an *in vivo* subcutaneous catheter rat model and on mucosal surfaces in the model of reconstituted human epithelium [36].

Similarly, the transcription factor *bcr1* that governs the adhesion process mediated by *als3* [34], the filamentation mediated by *hwp1* [37], and the development of persister cells in biofilm [38] was up-regulated at 8 hpi in our study in biofilm strains. The *mkc1* gene is expressed during the adhesion phase and is involved in contact-induced invasive filamentation, as well as in the maturation of biofilms [8]. This gene was up-regulated in biofilm strain at very early (4 hpi) and early (8 hpi) time points and the non-biofilm strain at 4 hpi, suggesting its role in early adhesion and cell proliferation.

Maximum up-regulation of genes related to adherence and filamentation was at 8 hpi, implying that the adhesion and initiation of the biofilm complex occur at most at 8 hpi. At later time points, host immunity may perhaps be able to control the burden of *Candida* and therefore the reduced expression of adherence- and filamentation-related genes and at the later timepoints, that is, 24 and 96 hpi. A study using *C. albicans* as a pathogen in the zebrafish model (liver) showed early 2 hpi as the adhesion phase, 2–8 hpi as the invasion phase, and beyond 12 hpi as the damage phase, irrespective of biofilm formation [20]. The discrepancy of survival between biofilm-forming and non-biofilm-forming zebrafish larvae may be influenced by a mixture of *Candida* morphotypes, that is, yeast and filamentous forms, while the non-biofilm-forming strain has only the yeast form [39].

In this study, three chosen strains possessed strong hemolysis activity, while other virulence activities, that is, phospholipase and proteinase activities, were negative. The hemolytic capability of *Candida* species is considered a virulence attribute as it can acquire host iron, facilitating cellular growth. In addition, *C. albicans* exploited iron from intracellular ferritin to promote biofilm formation though the hyphal associated adhesin and invasin (Als3), whose expression increased in strong biofilm former strains [40]. This interaction may contribute to virulence in our strong biofilm former strain.

In summary, our study emphasized that biofilm formation at the early phase with the hemolysin activity appeared to somewhat enhance the virulence of *C. albicans* in zebrafish. However, the association between biofilm-forming strength and *in vivo* virulence remains controversial. Genes related to adhesion and filamentation were also associated with biofilm-forming strength. For further study, gene expression related to biofilm associated with fungal metabolism and the study related to immune gene expression in the corresponding time frame of biofilm formation would be helpful in the demonstration of immune pathogenesis in the zebrafish model of infection.

## 5. Conclusions

The formation of biofilm by *C. albicans* is a strong strategy applied by the fungus to increase infectivity and mortality in the host, which is facilitated by earlier and higher up-regulation of genes related to biofilm formation. In practice, biofilm evaluation could be a good predictor of clinical outcomes in patients.

## Figures and Tables

**Table 1:** Primers and their sequences used in this study.

Sequence Name	Gene Function	Sequence (5′-3′)	Ta (°C)	References
*Act1*	Housekeeping gene (Internal control)	F: GCTGGTAGAGACTTGACCAACCA R: GACAATTTCTCTTTCAGCACTAGTAGTGA	54	[27]
*Eap1*	Cell–cell adhesion, mediates adhesion to biotic and abiotic surfaces	F: CTGCTCACTCAACTTCAATTGTCG R: GAACACATCCACCTTCGGGA	54	[27]
*Als3*	Mediates attachment to host cells and matrix proteins	F: GGTTATCGTCCATTTGTTGA R: TTCTGTATCCAGTCCATCTT	54	[28]
*Hwp1*	Cell adherence	F: ACAGGTAGACGGTCAAGG R: GGGTAATCATCACATGGTTC	50	[28]
*Bcr1*	Transcription factor controls the *als1*, *als3*, *hwp1*, and *hyr1* expressions	F: AATGCCTGCAGGTTATTTGG R: TTTTAGGTGGTGGTGGCAAT	50	[29]
*Mkc1*	Induced invasive filamentation and cell growth under stressed condition	F: AATGGGTCCAAAAAGGTTCC R: TTATGGCCCCTGAAGAACTG	50	This study

**Table 2:** Determination of *in vitro* virulence factors.

*C. albicans* Strains Used	Phospholipase Activity		Proteinase Activity		Hemolytic Activity		Biofilm Activity	
	Pz Value	Interpretation	Pr Value	Interpretation	Hz Value	Interpretation	OD	Interpretation
No. 104	1.00	**-**	1.00	**-**	0.59	**++**	0.031	No
No. 57	1.00	**-**	1.00	**-**	0.52	**++**	0.073	Weak
No. 140	1.00	**-**	1.00	**-**	0.53	**++**	0.396	Strong

Pz, Pr, Hz values were calculated by the ratio of colony diameter to the diameter of activity zone produced by phospholipase, proteinase, and hemolytic enzymes on the tested media, respectively; **-**, no activity; **++**, strong activity; and OD, optical density, measured at 450 nm and subtracted background at 630 nm using cut-off = 0.064.

## Data Availability

Not applicable.
